# Supplementary figures and images for: Antibody affinity versus dengue morphology influences neutralization
Source: PLoS Pathog. 2021 Feb 23;17(2):e1009331. doi: 10.1371/journal.ppat.1009331 (PMC7935256; doi:10.1371/journal.ppat.1009331)

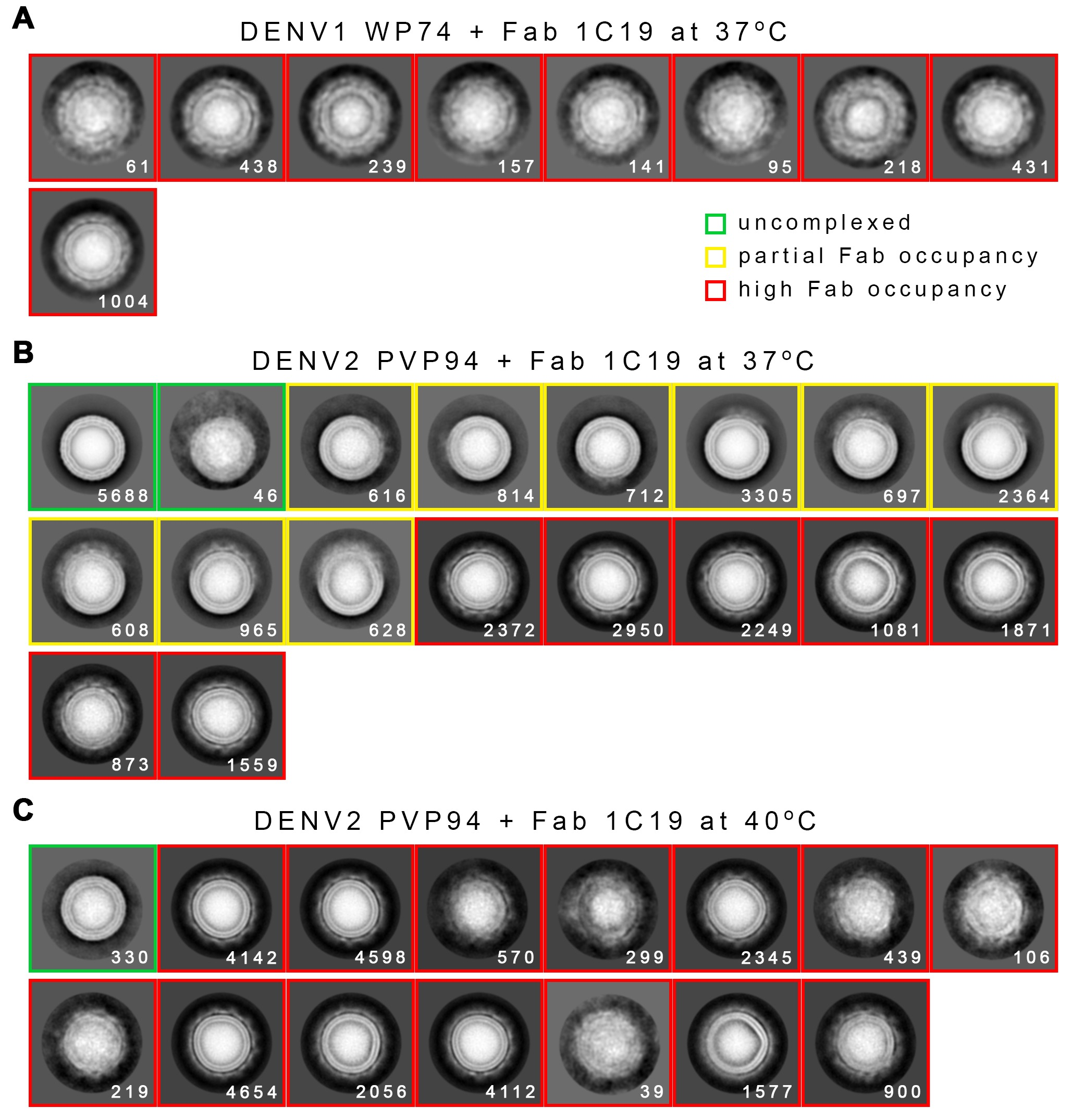

Supplement: S1 Fig — (A) The 2D class averages of DENV1 strain WestPac 74-Fab 1C19 complex particles at 37°C. All classes showed particles with likely high Fab 1C19 occupancy (red box) as indicated by the rough virus surfaces. The number of particles in each 2D classes is indicated. (B) The 2D class averages of DENV2 PVP94/07-Fab 1C19 complex particles at 37°C. The images showed heterogeneous populations of the complex particles at various occupancies—from uncomplexed (green) to partial (yellow box) or high occupancies. (C) The 2D class averages of DENV2 strain PVP94/07-Fab 1C19 complex particles at 40°C. Most classes showed particles with bound Fab 1C19 at high occupancy, and only a few unbound virus particles were left in the population. (TIF) [file ppat.1009331.s001.tif]

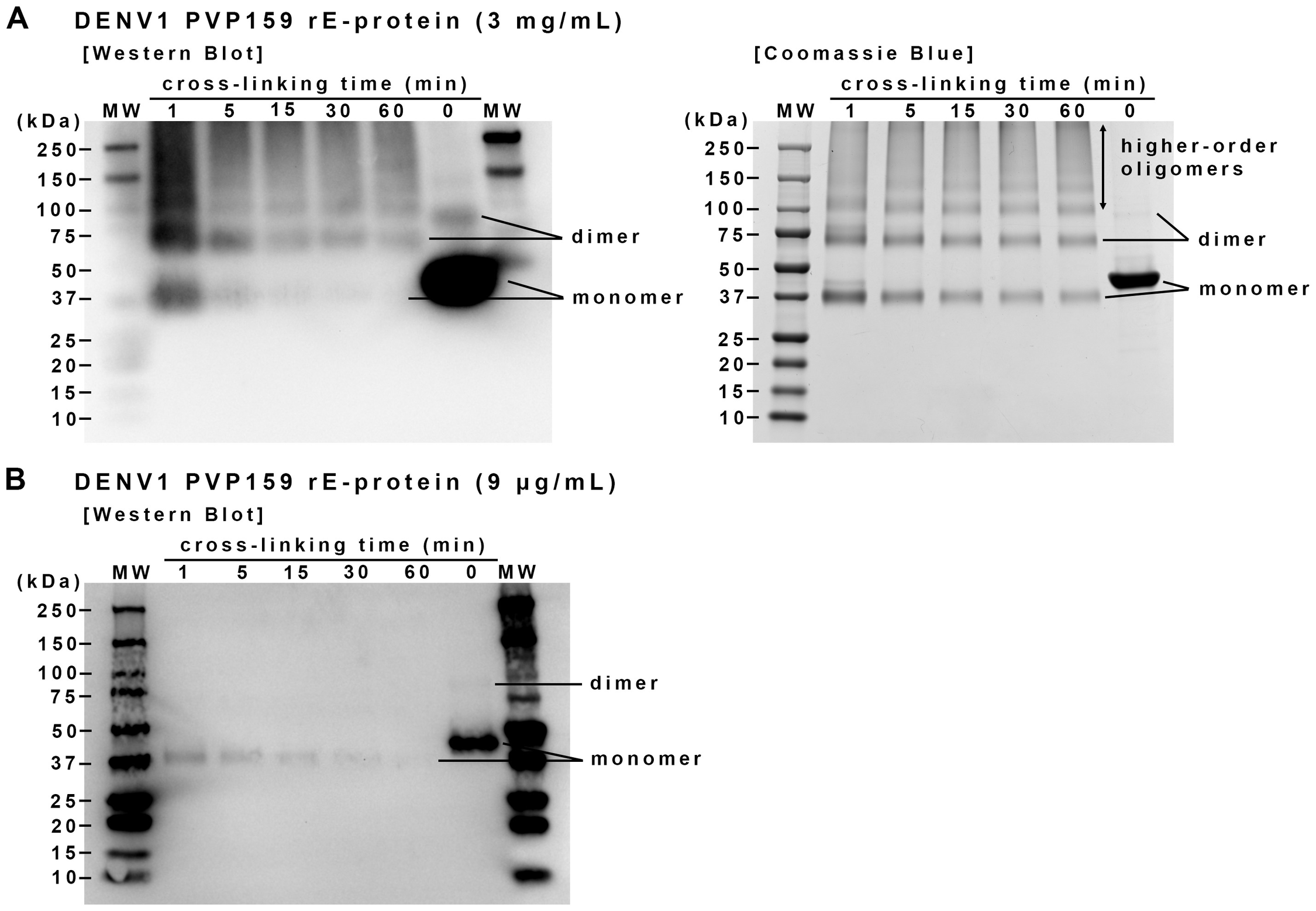

Supplement: S2 Fig — (A) Cross-linked DENV1 PVP159 rE protein concentration at 3 mg/mL similar to the that used in HDXMS experiments. Visualization of the E protein bands on non-reducing SDS-PAGE gel by either Western blot using HMAb 1C19 (left) or by staining with Commasie blue (right). Results show the presence of monomeric, dimeric and higher oligomeric states of E proteins in the protein sample at 3mg/mL and HMAb 1C19 is able to detect these different oligomerization states. (B) Similar crosslinking experiment is also done with DENV1 rE protein concentration at 9 μg/mL similar to that used in BLI experiments. At this concentration, E protein exists as monomeric state. The SDS-PAGE experiments were done in non-reducing condition (not boiled and without reducing agent eg. DTT or β-mercaptoethanol). (TIF) [file ppat.1009331.s002.tif]

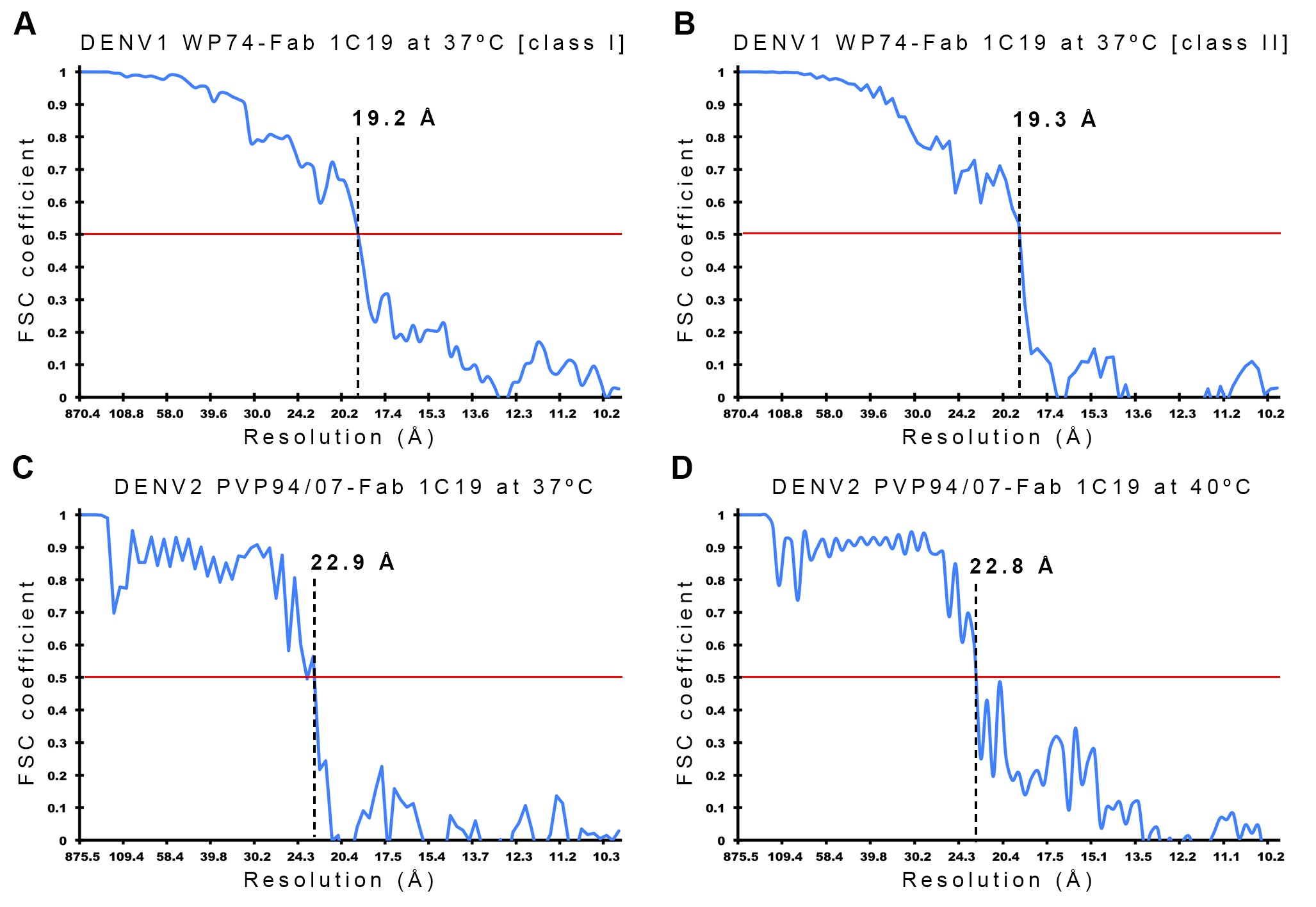

Supplement: S3 Fig — Fourier Shell Correlation (FSC) curve of: (A) Class I map of DENV1 strain WestPac 74-Fab 1C19 complex particles at 37°C. (B) Class II map of DENV1 strain WestPac 74-Fab 1C19 complex particles at 37°C. (C) Map of DENV2 strain PVP94/07-Fab 1C19 complex particles at 37°C. (D) Map of DENV2 strain PVP94/07-Fab 1C19 complex particles at 40°C. The FSC curve was plotted from two reconstructed maps of two half-datasets of the final iteration step and the resolution was estimated by using a 0.5 cut-off value. (TIF) [file ppat.1009331.s003.tif]

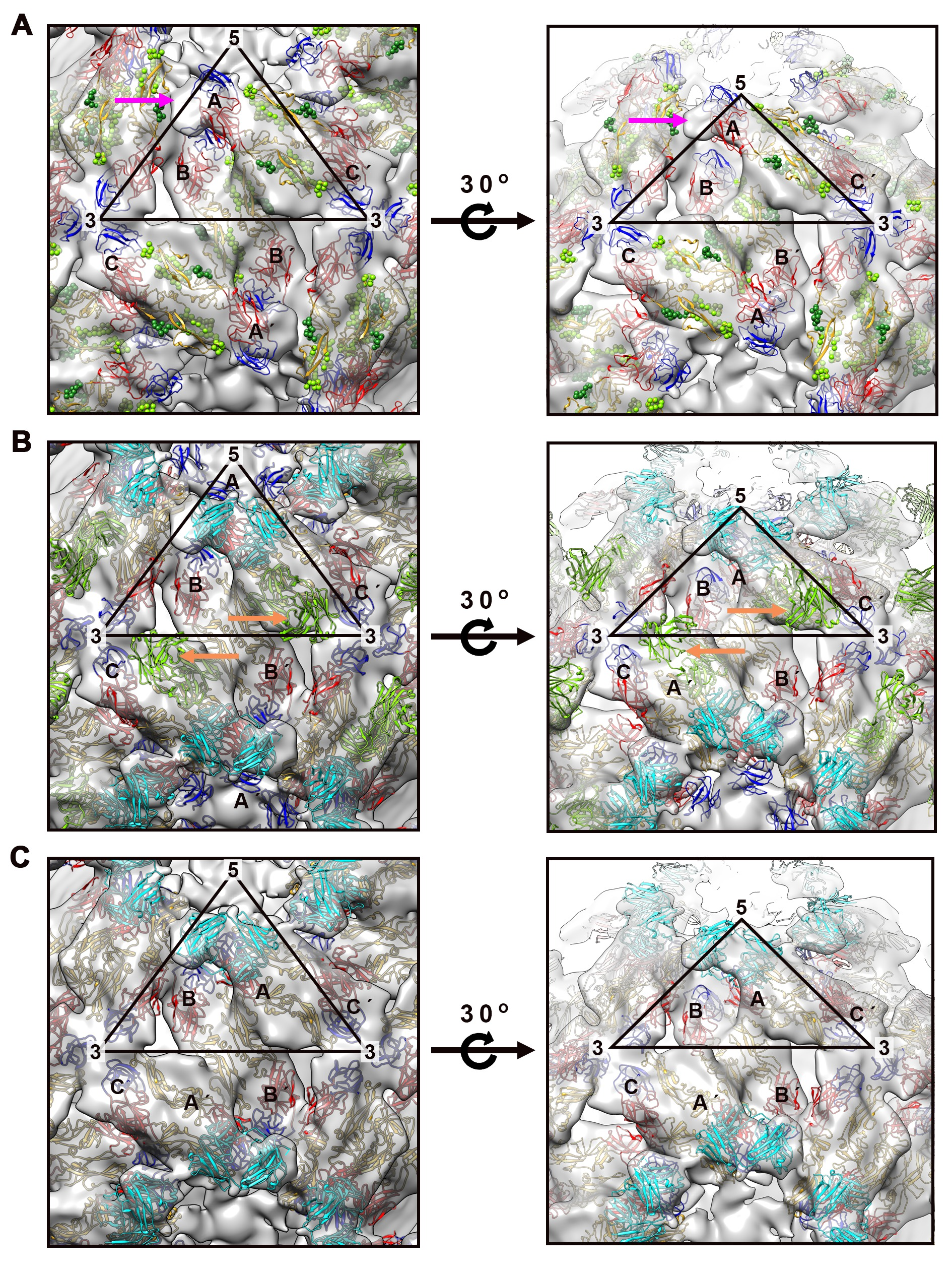

Supplement: S4 Fig — (A) The initial fitting was done by fitting the E protein mols B-B′ dimer into the density located on the 2-fold vertices and followed by fitting the E protein mols A-C′ dimer into the density located at a higher radius. The protruding density that belongs to the Fab 1C19 density, which is not occupied by the E protein mols A-C′ dimer, is indicated by a magenta arrow. (B) The E protein from the E protein mol A-Fab 1C19 model of Class I complex structure was superimposed onto both mols A and C′ E proteins, and then each of the E protein molecule-Fab 1C19 complex models was fitted as a rigid body separately into the density. Part of Fab 1C19 binding to E protein mol A is outside the density (indicated by orange arrows), suggesting that the Fab may not be present. (C) The final fit of the E proteins with Fab bound to only mol C into the density map is shown. (TIF) [file ppat.1009331.s004.tif]

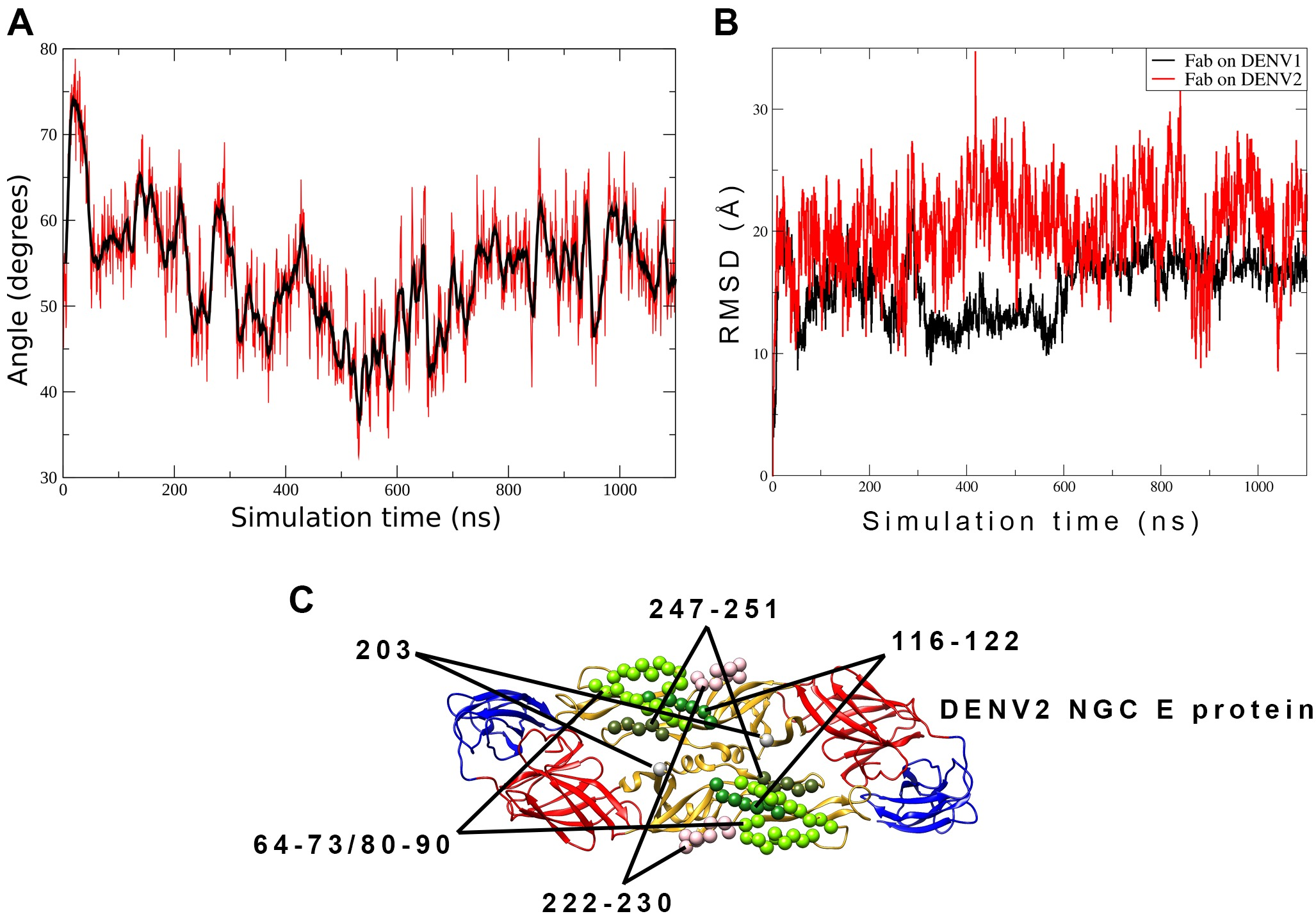

Supplement: S5 Fig — (A) The angle between the E protein dimer plane and the Fab long-axis vector over the simulation time for DENV1. (B) Root-mean-square-deviation (RMSD) of Fab backbone atoms on DENV1 and DENV2 dimeric E protein; this indicates that the DENV2 complex is less stable. (C) Epitope of 1C19 Fab on DENV2 E protein dimer. The top view protein is shown in cartoon representation (domain I: red, domain II: yellow, domain III: blue) with distinctive Fab interacting regions (shown as spheres). (TIF) [file ppat.1009331.s005.tif]

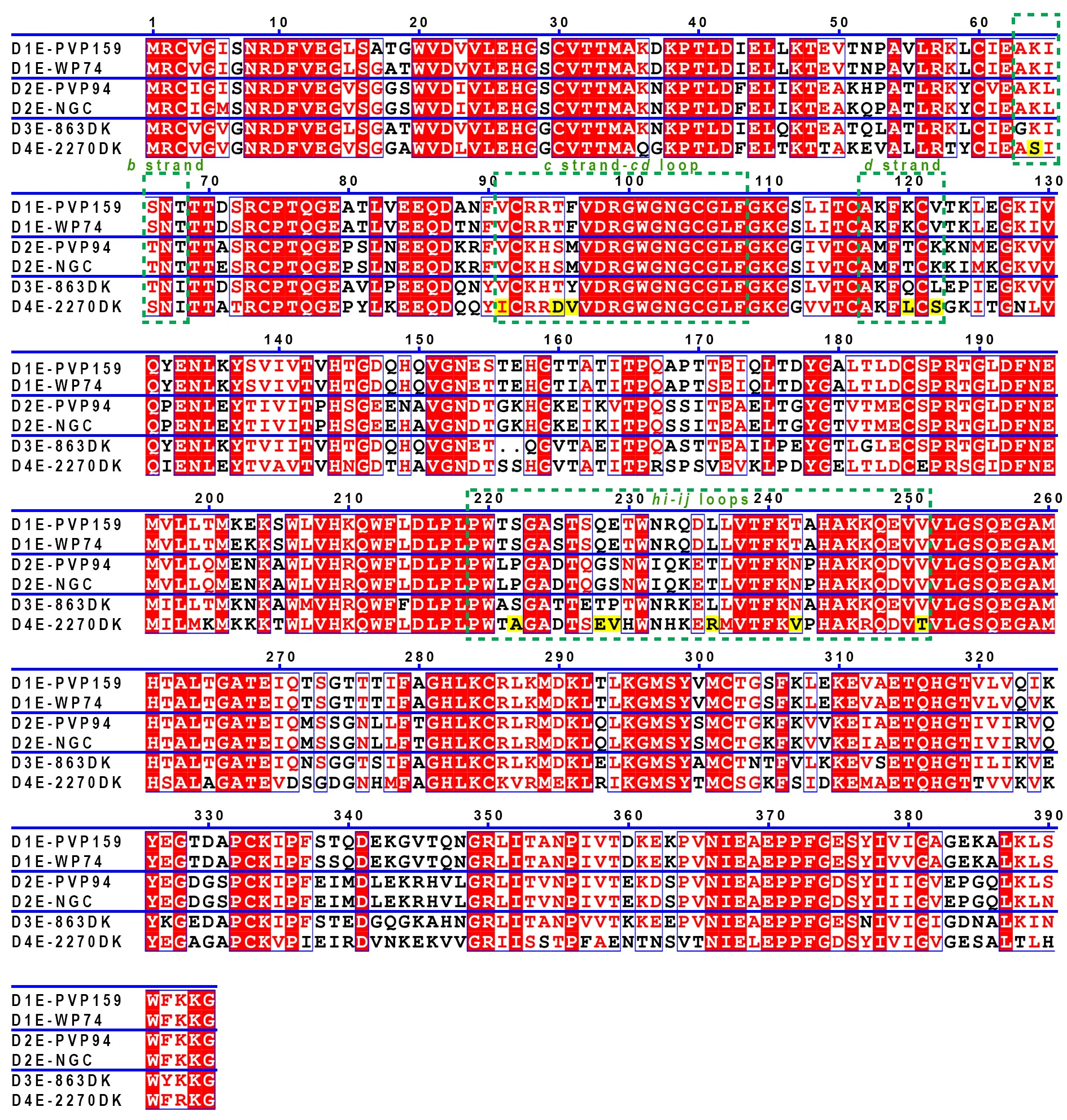

Supplement: S6 Fig — The amino acid residues on b strand, bc loop-c strand-cd loop and d strands, and hi and ij loops regions, which are estimated to be the location of the epitope of HMAb 1C19, are indicated in a box outlined with dashed green. Conserved amino acids are shown as letters in white font with red background, whereas partially conserved amino acids are shown as letters in red font with white background. This sequence alignment shows that the amino acid residues in the epitope region are highly conserved for different strains within the same serotype, but there are some differences across the two serotypes that may lead to different binding affinity to HMAb 1C19. (TIF) [file ppat.1009331.s006.tif]

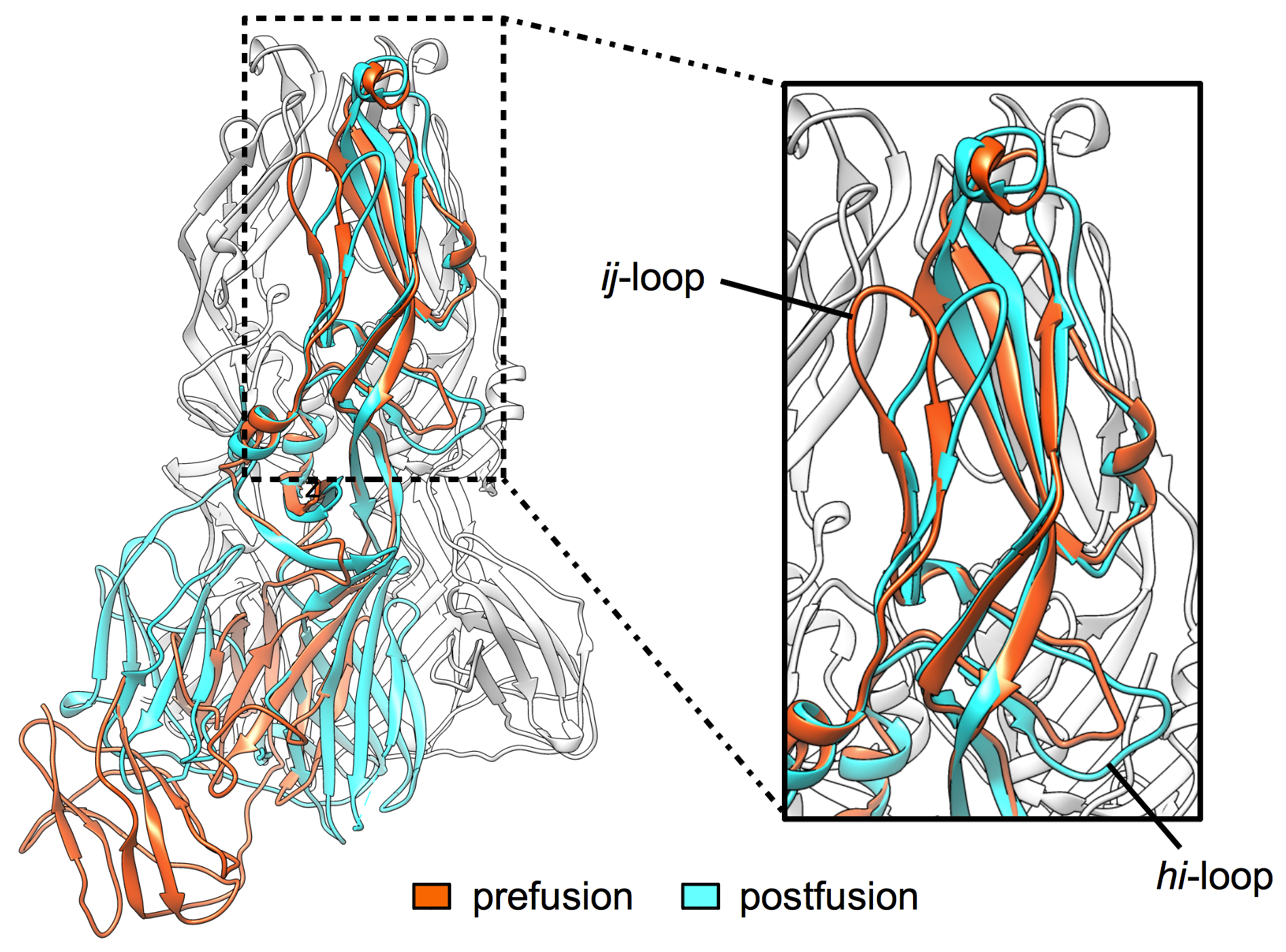

Supplement: S7 Fig — The superposition showed that the trimeric post-fusion E protein (one of which is colored in cyan, while the other molecule in grey) has a vastly different ij-loop conformation compared to the pre-fusion E protein (orange). The hi-loop also showed a different conformation. These features are both part of the 1C19 epitope identified by HDXMS. Both hi and ij loops are indicated. (TIF) [file ppat.1009331.s007.tif]

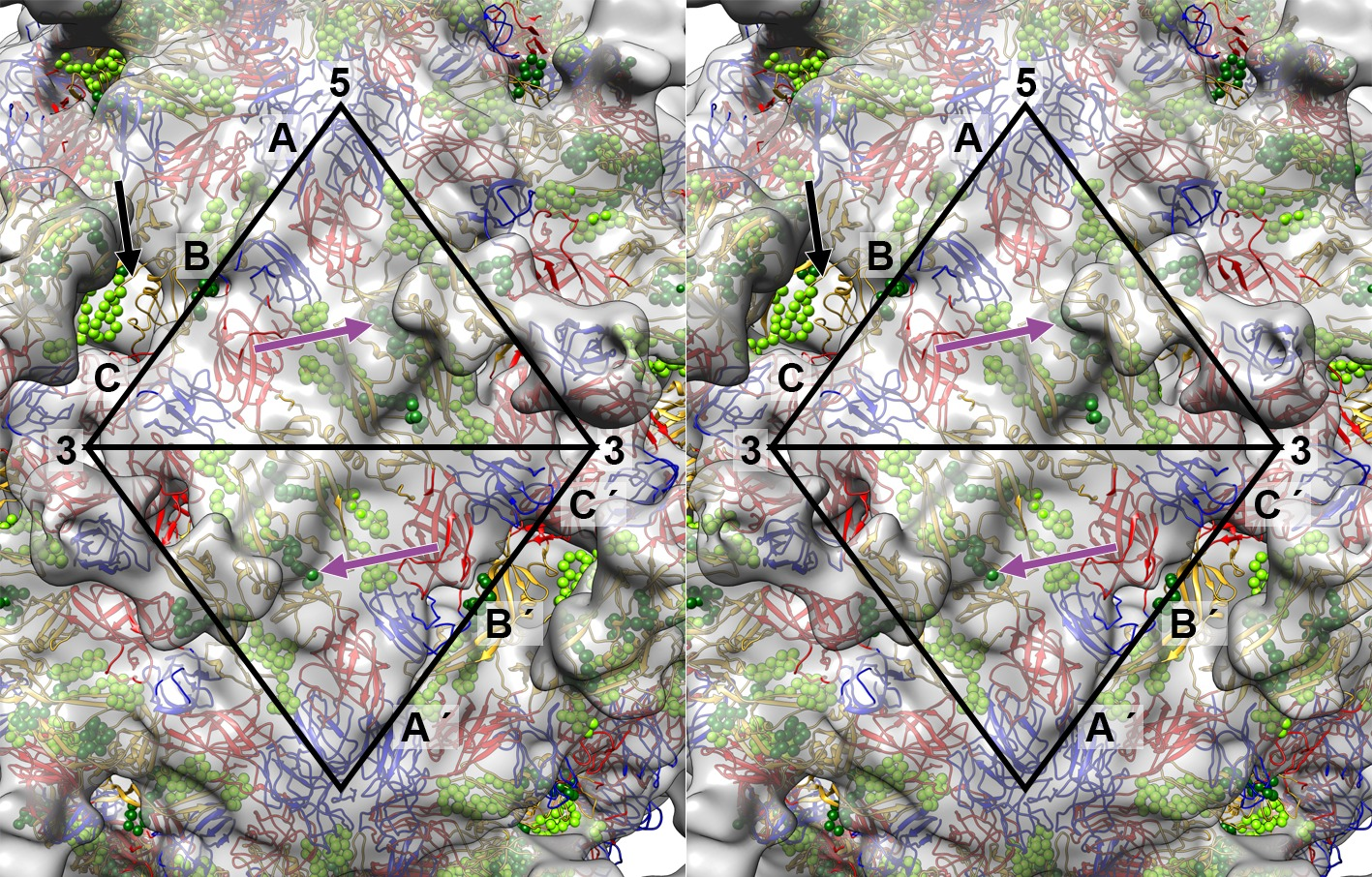

Supplement: S8 Fig — The unexpanded mature DENV1 structure (PDB ID 4CCT) in the Class I Fab 1C19:DENV1 WestPac complex map before fitting. The location of the putative epitope (green spheres) on all E protein molecules identified by HDXMS. Peptide 219–243 (green spheres), which was identified as contributing to the epitope by HDXMS studies, is located closest to the base of the Fab densities (purple arrow). Therefore the antibody most likely binds to mol A. However, some slight translation of the mol A was required in order to place the epitope at the foot of the antibody. Part of mol C is located in negative densities (black arrows) and therefore, some adjustments of position were required. Three individual E protein molecules in an asymmetric unit are labelled as A, B and C, whereas the corresponding molecules in a neighboring asymmetric unit within a raft are labelled as A′, B′ and C′. DI, DII or DIII of the E protein are colored in red, yellow or blue, respectively. (TIF) [file ppat.1009331.s008.tif]
