# Supplementary material for: Antibody affinity versus dengue morphology influences neutralization
Source: PLoS Pathog. 2021 Feb 23;17(2):e1009331. doi: 10.1371/journal.ppat.1009331 (PMC7935256; doi:10.1371/journal.ppat.1009331)
Supplement: S1 Table — (PDF) [file ppat.1009331.s009.pdf]

	Correlation coefficient	Average map value
**Class I**		
1 Fab	0.80	3.03
**Class II**		
1 Fab	0.77	2.61
2 Fabs	0.75	2.35
