# Supplementary material for: Antibody affinity versus dengue morphology influences neutralization
Source: PLoS Pathog. 2021 Feb 23;17(2):e1009331. doi: 10.1371/journal.ppat.1009331 (PMC7935256; doi:10.1371/journal.ppat.1009331)
Supplement: Attachment — Submitted filename: Fibriansah et al.docx [file ppat.1009331.s012.docx]

Fibriansah et al. use a series of complementary approaches to determine where the antibody 1C19 binds to the dengue E protein. They use two strains of DENV1 and two strains of DENV2 for their studies since these viruses bind to the antibody differently at 37C. The authors compare and contrast the results from each method. The work provides a basis for designing antibodies that can be used as therapeutics during dengue infection.

Comments.

For the two classes of structures determined by cryoEM, are the authors proposing these occur individually? Or Class II is an intermediate on the way to achieving Class I? Or vice versa, Class I occurs and induces the change seen in Class II?

The authors conclude antibody binding to E is a dynamic process and requires several discrete conformational changes by both the antibody and the E protein. Can the authors propose a model of how the particles move or undergo structural transitions based on their data? For example, using the data from DENV1 can the authors propose how 1C19 binds to DII, when it might interact and then dissociate with DIII, how E moves and how that may neutralize infection? The authors address parts of this question throughout the manuscript, I think it would beneficial if the authors could propose 1 (or 2 or 3) models of what might be happening.

When fitting the E protein into the cryoEM maps of DENV1, did the E protein monomer have to be rotated at all, thus exposing different residues compared to its conformation when a dimer?

Was the rE protein used for BLI and HDXMS experiments a monomer or a dimer or a mixture? If the E was a monomer, there are fewer steric constraints than if a dimer and higher likelihood for Fab to bind to the protein. In contrast, when an Fab binds to an E protein in a virus, a slight conformational change in one protein may have a more profound effect on neighboring E proteins because of the close packing. This was evident in the cryo structures. I also ask about this because HDXMS showed residues 219-243 as being the main Fab footprint. However, the cryo structures don’t directly show this footprint (near) and the MD simulations identify 239-252, slightly different.

Have the authors mutated the residues from DENV4 into DENV1 to see if the slight changes in surface residues is enough to reduce antibody binding?

The concluding two paragraphs of the discussion really emphasize the rationale of this work and the potential therapeutic benefits. I would like to see that brought up in the introduction. Maybe some mention of dengue treatments and why antibody treatment is worth pursuing.
