# Supplementary material for: Antibody affinity versus dengue morphology influences neutralization
Source: PLoS Pathog. 2021 Feb 23;17(2):e1009331. doi: 10.1371/journal.ppat.1009331 (PMC7935256; doi:10.1371/journal.ppat.1009331)
Supplement: Attachment — Submitted filename: reviewers_comments_Jan17.docx [file ppat.1009331.s013.docx]

Dear Dr. Lok,

Thank you very much for submitting your manuscript "Antibody affinity versus dengue morphology influences neutralization" for consideration at PLOS Pathogens. As with all papers reviewed by the journal, your manuscript was reviewed by members of the editorial board and by several independent reviewers. The reviewers appreciated the attention to an important topic. Based on the reviews, we are likely to accept this manuscript for publication, providing that you modify the manuscript according to the review recommendations.

The manuscript was reviewed by three individuals with expertise in the structure and biology of dengue virus and the challenges faced in developing dengue virus vaccines. Overall, all the three reviewers were quite positive in their assessment of the work, commenting favorably on the nature of the experimental approach and the significance of the findings. However, reviewers did raise issues in a few areas that need to be addressed. For example, (i) Reviewer 2 was not convinced of the inference brought up in the Discussion that the 1C19 antibody could serve as an effective therapeutic, given the results of previous studies suggesting the opposite, (ii) Reviewer 1 wanted additional information regarding the interpretation of the cryoEM work, and (iii) Reviewer 1 also thought key details were missing for the BL1 and HDMXS experiments that are necessary for analyzing the their results. Although these are generally minor issues, they will need to be thoroughly addressed and the manuscript appropriately modified before further consideration for publication. Thank you again for submitting such an outstanding manuscript to the journal. We look forward to receiving the revised manuscript in the near future and its ultimate publication.

Please prepare and submit your revised manuscript within 30 days. If you anticipate any delay, please let us know the expected resubmission date by replying to this email.

When you are ready to resubmit, please upload the following:

[1] A letter containing a detailed list of your responses to all review comments, and a description of the changes you have made in the manuscript.

Please note while forming your response, if your article is accepted, you may have the opportunity to make the peer review history publicly available. The record will include editor decision letters (with reviews) and your responses to reviewer comments. If eligible, we will contact you to opt in or out

[2] Two versions of the revised manuscript: one with either highlights or tracked changes denoting where the text has been changed; the other a clean version (uploaded as the manuscript file).

Important additional instructions are given below your reviewer comments.

Thank you again for your submission to our journal. We hope that our editorial process has been constructive so far, and we welcome your feedback at any time. Please don't hesitate to contact us if you have any questions or comments.

Sincerely,

John T. Patton, PhD
Associate Editor
PLOS Pathogens

Mark Heise
Section Editor
PLOS Pathogens

Kasturi Haldar
Editor-in-Chief
PLOS Pathogens
orcid.org/0000-0001-5065-158X

Michael Malim
Editor-in-Chief
PLOS Pathogens
orcid.org/0000-0002-7699-2064

***********************

Thank you very much for submitting your manuscript "Antibody affinity versus dengue morphology influences neutralization" (PPATHOGENS-D-20-02220) for consideration by PLOS Pathogens. The manuscript was reviewed by three individuals with expertise in the structure and biology of dengue virus and the challenges faced in developing dengue virus vaccines. Overall, all the three reviewers were quite positive in their assessment of the work, commenting favorably on the nature of the experimental approach and the significance of the findings. However, reviewers did raise issues in a few areas that need to be addressed. For example, (i) Reviewer 2 was not convinced of the inference brought up in the Discussion that the 1C19 antibody could serve as an effective therapeutic, given the results of previous studies suggesting the opposite, (ii) Reviewer 1 wanted additional information regarding the interpretation of the cryoEM work, and (iii) Reviewer 1 also thought key details were missing for the BL1 and HDMXS experiments that are necessary for analyzing the their results. Although these are generally minor issues, they will need to be thoroughly addressed and the manuscript appropriately modified before further consideration for publication. Thank you again for submitting such an outstanding manuscript to the journal. We look forward to receiving the revised manuscript in the near future and its ultimate publication.


Reviewer Comments (if any, and for reference):

Reviewer's Responses to Questions

**Part I - Summary**
Please use this section to discuss strengths/weaknesses of study, novelty/significance, general execution and scholarship.

Reviewer #1: Fibriansah et al. use a series of complementary approaches to determine where the antibody 1C19 binds to the dengue E protein. They use two strains of DENV1 and two strains of DENV2 for their studies since these viruses bind to the antibody differently at 37C. The authors compare and contrast the results from each method. The work provides a basis for designing antibodies that can be used as therapeutics during dengue infection.

We thank reviewer #1 for the positive comment.

Reviewer #2: Human MAb 1C19 was isolated previously from a secondary dengue patient. This antibody binds to an epitope that is conserved between serotypes. Moreover, the antibody cross neutralizes serotypes 1-3 in cell culture. In this manuscript, Fibriansah et al map the binding site of 1C19 on dengue type 1 and 2 using deuterium exchange spectroscopy and cryo EM. The results demonstrate that the antibody binds a complex cryptic epitope on domain II of E protein. The exposure of the epitope was dependent on temperature, the morphology of the virus and the serotype. The strengths of the paper are the excellent and complementary approaches used to map the epitope and the design of studies to link viral envelope dynamics to antibody binding.

We thank the reviewer #2 for the positive comment.

The main weakness of the paper is a superficial discussion of how this antibody might be used as a therapeutic antibody. The studies reported here as well as previous work with this antibody strongly argue against any useful role for this (and related antibodies) in protection as discussed below.

Thank you for pointing this out. We found the same in our neutralization test and cryoEM studies that the antibody is only neutralizing to dengue 2 particles when they adopt a bumpy morphology, and we have previously found that clinical dengue 2 strains [1,2] are mostly smooth compact surface and thus likely not susceptible to 1C19. We have never indicated in the paper that 1C19 is a good therapeutic candidate (in fact in agreement with reviewer #2, we think otherwise). But by studying this antibody, it gives us a hint as to what a good therapeutic antibody should be like (high affinity). We will answer this question in detail below.

1. Lim XN, Shan C, Marzinek JK, Dong H, Ng TS, Ooi JSG, Fibriansah G, Wang J, Verma CS, Bond PJ, Shi PY, Lok SM. PLoS Pathog. 2019 Sep 19;15(9):e1007996.
2. Fibriansah G, Ibarra KD, Ng TS, Smith SA, Tan JL, Lim XN, Ooi JS, Kostyuchenko VA, Wang J, de Silva AM, Harris E, Crowe JE Jr, Lok SM. Science. 2015 Jul 3;349(6243):88-91.

Reviewer #3: This work characterizes the neutralizing human mAb 1C19 for four strains belonging to two dengue serotypes, DENV1 and DENV2. The authors mapped the 1C19 epitope to the DENV E protein domain II using hydrogen-deuterium exchange mass spectrometry, cryoEM, and molecular dynamics. The 1C19 binds to DENV1 E protein with higher affinity compared to that of DENV2, and displays strong neutralizing activities for all DENV1 strains regardless of their morphologies, suggesting the tighter binding of 1C19 can outcompete the E-E interactions. This study is well-executed and provides structural and mechanistic insights into the neutralization of DENV by a human mAb.

We thank reviewer #3 for the positive comments.

**Part II – Major Issues: Key Experiments Required for Acceptance**
Please use this section to detail the key new experiments or modifications of existing experiments that should be absolutely required to validate study conclusions.

Generally, there should be no more than 3 such required experiments or major modifications for a "Major Revision" recommendation. If more than 3 experiments are necessary to validate the study conclusions, then you are encouraged to recommend "Reject".

Reviewer #1: For the two classes of structures determined by cryoEM, are the authors proposing these occur individually? Or Class II is an intermediate on the way to achieving Class I? Or vice versa, Class I occurs and induces the change seen in Class II?

Class I structure has antibody binding to E protein mol. A and Class II, mol C. We have never observed a structural class that contains antibodies binding to both mols A and C. Hence we conclude that the two classes (Class I and II) occur separately. We have edited the discussion to emphasize this point:

“The DENV1 WestPac 74-Fab 1C19 Class II and the DENV2 PVP94-Fab 1C19 structures at 37°C are similar to each other, and they showed a different binding mode with Fab 1C19 compared to that in the DENV1 WestPac 74-Fab 1C19 Class I map (Fig 4C and 4D). The Fab 1C19 binds to the E protein mol C in DENV1 WestPac 74-Fab 1C19 Class II and the DENV2 PVP94-Fab 1C19 at 37°C structures, whereas in the DENV1 WestPac 74-Fab 1C19 Class I, mol A is bound (Fig. 5). These data suggest that the Fab 1C19 can only bind to either one of the protomers in the mol A/C′ dimer, depending on which is exposed first during the E protein movement at 37°C, hence the two classes do not represent the sequence of occurrence but rather two individual states. On the other hand, the B/B′ dimer in all of our maps did not move much from the original radius in the compact smooth particles and Fabs did not bind to this dimer.”

The authors conclude antibody binding to E is a dynamic process and requires several discrete conformational changes by both the antibody and the E protein. Can the authors propose a model of how the particles move or undergo structural transitions based on their data? For example, using the data from DENV1 can the authors propose how 1C19 binds to DII, when it might interact and then dissociate with DIII, how E moves and how that may neutralize infection? The authors address parts of this question throughout the manuscript, I think it would beneficial if the authors could propose 1 (or 2 or 3) models of what might be happening.

To understand more about how 1C19 binds better to DENV1 than DENV2, we conducted MD simulations of 1C19 binding to both viruses and showed that 1C19 probably has a bigger footprint on DENV1 than DENV2 E protein hence explaining the higher affinity of 1C19 towards DENV1.

We included Fig. S5, and have written about this in the Discussion (paragraph 6):

“We considered whether this difference in neutralization could also be affected by differences in surface residues within the epitope between strains, leading to reduced antibody binding. To resolve this question, we conducted biolayer interferometry measurements to compare binding of HMAb 1C19 to recombinant E proteins of different strains within a serotype (Fig 2B). HMAb 1C19 binds to E proteins of DENV1 PVP159 and WestPac 74 strains at subnanomolar affinity (0.71 and 0.84 nM, respectively) (Fig. 2A-B). Whereas, the affinity to E protein of DENV2 PVP94/07 and NGC strain are lower at nanomolar range (19.02 and 4.55 nM, respectively) (Fig. 2C-D). The affinity differences between DENV1 and DENV2 strains can be seen from the dissociation rate, which is steeper for E proteins of DENV2 strains.

To understand why the affinity of 1C19 is lower for binding DENV2 E protein, we conducted MD simulations of DENV1 and DENV2 E proteins complexed with Fab 1C19.

For DENV2 MD simulations, we used the cryoEM DENV1:Fab 1C19 complex structure but we substituted the E protein sequence with that of the DENV2 NGC strain (S5C Fig).

The simulations revealed that Fab on DENV2 was more dynamic than the Fab on DENV1 (S5B Fig), and there are likely only 43 interacting residues on DENV DII E protein, about half of those on DENV1 (79 residues). Also, Fab 1C19 is not likely to have any interaction with DIII on DENV2 E protein (S5C Fig) compared to the transient interactions on DENV1 (Fig. 6C).”

**“Fig.** **S5. MD simulations of DENV1 and DENV2 NGC strain interacting with Fab 1C19. (A)** The angle between the E protein dimer plane and the Fab long-axis vector over the simulation time for DENV1. **(B)** Root-mean-square-deviation (RMSD) of Fab backbone atoms on DENV1 and DENV2 dimeric E protein; this indicates that the DENV2 complex is less stable. **(C)** Epitope of 1C19 Fab on DENV2 E protein dimer. The top view protein is shown in cartoon representation (domain I: red, domain II: yellow, domain III: blue) with distinctive Fab interacting regions (shown as spheres).”

As reviewer has suggested, we also included extra information in the Discussion section (paragraph 9) and included a new figure (Fig 7) on the models of how 1C19 binds and neutralize viruses:

“These structural findings provide a better understanding of how the antibody neutralizes the virus once it achieves sufficient occupancy on the virus surface. Part of the epitope of HMAb is located on *hi-ij* loops, which is near to the glycosylation site on residue N67 (Fig 6A). The N67 residue is important for binding to the DC-SIGN receptor [35], and hence HMAb 1C19 binding to the epitope may prevent this interaction. In addition, HMAb 1C19 may also block fusion. Comparison of the 4Å resolution structure of E protein of the mature DENV1 [36] to the crystal structure of the post-fusion DENV1 E protein showed that the *ij* loop, which is also part of the 1C19 epitope, has a very different conformation (S7 Fig). When the antibody binds to this loop, it may stop the structural changes needed to transition to the post-fusion structure, thus inhibiting the fusion process. In summary, we proposed models of how HMAb 1C19 could bind and neutralize viruses. (Fig 7). HMAb 1C19 mainly binds to a partially hidden epitope on E protein DII of either mol A or C - depending upon which is exposed first when the E proteins are “vibrating” at higher temperatures (37ºC or 40ºC). Once bound to the epitope, HMAb 1C19 can disrupt the quaternary structure of the virus, this may prevent the virus from attaching to the cell and/or blocking virus-endosomal membrane fusion.”

**“Fig. 7. Binding of Fab 1C19 to DENV1 and DENV2. Fab 1C19 binds to a partially hidden epitope on DII of the E proteins.** It has a strong affinity to DENV1 E protein and therefore it can out-compete E-to-E interactions even when the virus maintains a smooth compact surface that only partially reveals the epitope. 1C19 can only bind to one of the E proteins of the AC dimer, depending on which is exposed first when the E protein is vibrating at 37ºC. 1C19 binds in a similar same way to DENV2 (either mol A or C), but because its affinity to DENV2 epitope is lower, the virus has to change its conformation to a looser “bumpy” surface structure at higher temperatures (37ºC or 40ºC) to expose its epitope fully before the antibody can bind.”

Regarding the interaction of Fab 1C19 with DIII in DENV1, we think it does not form the major epitope. This is because, that will require the E protein to exist in dimers; if this is true, then we would expect the antibody is able to bind to the virus at 4ºC when the dimer is the least mobile. However, we observed the opposite – Fab 1C19 does not bind to the virus at 4ºC. The antibody tends to prefer to bind to looser virus when the E protein dimeric interaction is weaker, suggesting DIII binding to the opposite E protein is redundant. Also, our Fab 1C19:virus complex cryoEM map shows that the E protein protomers have separated- again if the Fab 1C19 also binds across the DIII of the opposite E protein, you would expect the E protein dimer structure to be preserved, but that is not what we have observed.

We have modified our discussion to include and clarify this:

“The MD and HDXMS results indicate that there are significant dynamic properties of the virion particle affecting Fab 1C19 binding to E protein, with the majority of the epitope located on DII of a protomer, but also that the antibody can bind transiently to DIII on the opposite protomer. MD simulations show that the constant region of Fab is involved in transient binding to DIII. This interaction likely does not form the main binding epitope. This is because binding of Fab across the E proteins requires the E protein to be in a stable dimeric structure; in this case, you would expect that the Fab would prefer to bind to virus at 4ºC where the dimers are the most stable. However, the opposite is observed, few or no Fabs are observed to bind to DENV1 or DENV2 particles at 4ºC. Also, in the cryoEM structure, the E protein protomers within the dimer had dissociated after Fab binding, suggesting that the DII residues on one protomer alone are likely sufficient for binding.”

When fitting the E protein into the cryoEM maps of DENV1, did the E protein monomer have to be rotated at all, thus exposing different residues compared to its conformation when a dimer?

This is shown in our manuscript (Fig 5), and also we have described in our Results section, how we fitted the molecules into the map. As the cryoEM map is low in resolution (19 Å), we conduct rough fitting of the E protein - we did translate and rotate (around the axis perpendicular to the E protein dimer) the E proteins. We did not rotate the E proteins along their long axis (parallel to the E protein dimer), as the resolution of the map did not permit us to do that. Even with this fitting, we can see the exposure of residues is definitely different compared to the dimer (Fig 5).

We have modified the Results section to emphasize this:

“To fit the E proteins and Fabs into their corresponding densities, we did translate and rotate some of the E proteins along the axis perpendicular to E protein dimer. The E protein and Fab models of the Class I cryo-EM map (Fig 4B) show that while the B-B′ dimer only moved very slightly from the original smooth compact virus structure, the mols A and C′ dimers are likely separated (Fig. 5, A and B). Hence, the solvent accessibility of the residues on mols A and C will be higher than in the former dimeric structure. The mols C are arranged underneath the B-B′ dimer, resulting in the presence of holes around the 3-fold vertices (Fig. 5A, middle panel). The fitting showed that Fab 1C19 likely interacts with DII of mol A (Figs. 4B and 5A). The epitope covered by the Fab molecule, as observed from the cryo-EM map of Class I particles (Fig 6A, left panel), overlaps with that identified by the HDXMS studies (Fig 6A, right panel). The low resolution of the Class I DENV1-Fab 1C19 map did not allow for precise analysis of the interacting residues. Electrostatic potential surface analysis showed complementarity between the cryoEM identified epitope and paratope (Fig 6B).”

Was the rE protein used for BLI and HDXMS experiments a monomer or a dimer or a mixture? If the E was a monomer, there are fewer steric constraints than if a dimer and higher likelihood for Fab to bind to the protein. In contrast, when an Fab binds to an E protein in a virus, a slight conformational change in one protein may have a more profound effect on neighboring E proteins because of the close packing. This was evident in the cryo structures.

To investigate the oligomeric state of rE protein used for BLI and HDXMS experiments, we have performed glutaraldehyde cross-linking experiments followed by SDS PAGE analysis (Coomassie blue stained and/or Western blot using HMAb 1C19). The E concentration for BLI is undetectable by SDS-PAGE, and hence only western blot is used. The western blot results showed that at low concentration (9 $\mu$g/mL, concentration used for BLI experiment), the rE protein exists as monomers, whereas at higher concentration (3 mg/mL), there is a mixture of monomers, dimers and higher oligomers. HMAb 1C19 seems to be able to bind to all soluble E protein oligomers. This suggests that the soluble E protein may be able to “bend” more than those on the virus surface, thereby allowing the epitope to become exposed.

We have included these glutaraldehyde cross-linking experiment results in their associated Results sections on BLI and HDXMS and the Discussion section.

In the Results section:

BLI section:

“We considered whether the differential binding patterns for DENV1 versus DENV2 particles we have observed were due to differences in affinity of binding of the antibody to DENV1 and DENV2 E proteins. We conducted biolayer interferometry (BLI) studies to determine the affinity of HMAb 1C19 for binding to recombinant DENV1 or DENV2 E proteins. The results showed that HMAb 1C19 has overall higher affinities (~5.4 to 26.7-fold higher) to DENV1 strains E proteins (K_D_= 0.712 and 0.844 nM for PVP159 and WestPac 74 strains, respectively) than to those of the DENV2 strains (K_D_= 19.015 and 4.549 nM for PVP94/07 and NGC strains, respectively) (Fig 2A-2D). To characterize the oligomeric state of the E protein used in BLI, a similar concentration of E protein (9 $\mu$g/mL) is used for glutaraldehyde cross-linking experiments. It is not possible to use Coomassie blue stained gel to visualize E protein bands as the concentration of E proteins is too low. Therefore, we conducted a western blot assay with HMAb 1C19 (which has been shown to be able to bind both monomeric and dimeric E protein on gel (S2A Fig, left)). The result showed that the E proteins at 9 $\mu$g/mL primarily exist in a monomeric state (S2B Fig).”

In the HDXMS section:

“Peptide 302-315 had a lower level of deuterium alteration of ~0.65 (Fig 3A and 3B). This finding suggests the binding of the Fab 1C19 may be very dynamic, such that Fab binding to this region could be transient. This peptide is likely located on DIII of the opposite E protein protomer within a dimer. This is because this peptide is located too far away from the primary binding site (peptide 219-243 with the highest deuterium alteration) on DII within a protomer. To investigate if there is indeed presence of E protein dimers in the HDXMS mixture, we prepared E protein at a concentration of 3 mg/mL (similar to that used for HDXMS) to conduct glutaraldehyde cross-linking experiments. Both Coomassie-stained SDS PAGE and also western blot analysis using 1C19 antibodies showed the presence of monomers, dimers and higher order oligomers (S2A Fig).”

I also ask about this because HDXMS showed residues 219-243 as being the main Fab footprint. However, the cryo structures don’t directly show this footprint (near) and the MD simulations identify 239-252, slightly different.

Regarding the correlation of the major footprint between HDXMS and MD, MD simulations are conducted over a timescale of 1.1 microseconds, which is long by MD standards but limited compared to experiment, where the closest HDXMS condition to this is the 1 min D_2_O incubation time. MD identified residues 239-252 (Fig. 6); for the HDXMS 1min D_2_O incubation, the highest altered deuterated peptide is 236 to 251 (Fig. 3A). They are actually highly correlated. As for the cryoEM fitting, we did also consider the information we obtained from MD and HDXMS when fitting the structure. We now include a new figure (Fig 6A, right) to show the top view of the fitting with the density and showed that peptide 236-251 is within the region where the Fab is bound.

We have now included in the discussion (paragraph 2) about the correlation of HDXMS with MD data:

“The epitope identified by HDXMS, cryoEM and MD correlates very well. MD simulations were performed over a timescale 1.1 μs (S5A Fig); the closest HDXMS condition to this is the 1 min D_2_O incubation time (Fig 3A). MD identified residues 239-252 (Fig. 6C), and the HDXMS at 1min D_2_O incubation shows that the highest altered deuterated peptide is between residues 236 to 251 (Fig. 3A). The cryoEM fit also shows that E protein residues 236-251 are within the region where the Fab is bound (Fig. 6A, middle).”

Have the authors mutated the residues from DENV4 into DENV1 to see if the slight changes in surface residues is enough to reduce antibody binding?

We have indicated in a sequence alignment of DENV1-4 (S6 Fig) where we have identified residues within the epitope that are different in DENV4 compared to DENV1-3; these are residues 64 (corresponding residues in DENV1/DENV4 is K/S), 91 (V/I), 95 (T/D), 96 (F/V), 120 (K/L), 122 (V/S), 222 (S/A), 228 (Q/E), 229 (E/V), 236 (L/R), 242 (T/V), and 251 (V/T). It is possible there will be either one mutation or a combination of mutants that will reduce antibody binding. Hence, we think the mutation study is out-of-scope for the current manuscript. However, it will be interesting to investigate this aspect of the epitope in the future.

We have now included this in the first paragraph of the discussion:

“Comparing the electrostatic charges of the 1C19 epitope between serotypes showed that DENV4 differs the most in terms of charge, which may explain the inability of HMAb 1C19 to neutralize DENV4. We also generated a sequence alignment of the epitopes across DENV1-4 (S6 Fig) to show that DENV4 has unique residues in the following positions: residues 64 (corresponding residues in DENV1/DENV4 is K/S), 91 (V/I), 95 (T/D), 96 (F/V), 120 (K/L), 122 (V/S), 222 (S/A), 228 (Q/E), 229 (E/V), 236 (L/R), 242 (T/V), and 251 (V/T).”

Reviewer #2:

While no additional experiments are required, I do not agree with the conclusion in the abstract and discussion that these studies support the use of this antibody as a therapeutic. The dengue field is currently contending with the fact that in vitro antibody neutralization is not a reliable predictor of protection in vivo. Indeed, this study highlights how dynamic properties of lab strains of dengue viruses can lead to neutralization in cell culture by mechanisms that have no relevance to protection in vivo.

1) In the original publication describing 1C19 (Smith et al), the antibody given prophylactically to mice had a modest effect on DENV1 (1 log reduction) and DENV2 (2.5 log reduction) viremia. This effect is mild compared to other human antibodies that provide close to sterilizing immunity when administered prophylactically.

2) Tuan et al compared a large panel of human MAbs for their ability to directly neutralize DENVs in blood collected from viremic dengue patients. In that study many antibodies such as 2D22, 14C10, EDE Mabs neutralized "in vivo" virus. 1C19 had no effect on any DENV serotype ( Tuan Vu T et al. (2019) Blockade of dengue virus transmission from viremic blood to Aedes aegypti mosquitoes using human
monoclonal antibodies. PLoS Negl Trop Dis 13(11): e0007142).

The investigators need to frame their work against this body of work as well as recent vaccine trials demonstrating cell culture neutralization is not always predictive of protection in vivo.

We thank reviewer for pointing this out. We agree that 1C19 is not suitable as a therapeutic candidate and our results support that. We did not anywhere in the paper say that 1C19 is a good therapeutic antibody, but our work does show what characteristics a good therapeutic antibody should encompass – high affinity, such that it can neutralize all dengue virus serotypes and morphology. This may not have been clear in the paper, and we have now modified the paper to point this out explicitly.

New abstract:

“Different strains within a dengue serotype (DENV1-4) can have smooth, or “bumpy” surface morphologies with different antigenic characteristics at average body temperature (37°C). We determined the neutralizing properties of a serotype cross-reactive human monoclonal antibody (HMAb) 1C19 for strains with differing morphologies within the DENV1 and DENV2 serotypes. We mapped the 1C19 epitope to E protein domain II by hydrogen deuterium exchange mass spectrometry, cryoEM and molecular dynamics simulations, revealing that this epitope is likely partially hidden on the virus surface. We showed the antibody has high affinity for binding to recombinant DENV1 E proteins compared to those of DENV2, consistent with its strong neutralizing activities for all DENV1 strains tested regardless of their morphologies. This finding suggests that the antibody could out-compete E-to-E interaction for binding to its epitope. In contrast, for DENV2, HMAb 1C19 can only neutralize when the epitope becomes exposed on the bumpy-surfaced particle. Although HMAb 1C19 is not a suitable therapeutic candidate, this study with HMAb 1C19 shows the importance of choosing a high-affinity antibody that could neutralize diverse dengue virus morphologies for therapeutic purposes.”

In the last paragraph of the discussion:

“*In vitro* work from Smith *et al*. (2013) [33] showed the neutralization capability of HMAb 1C19 to lab adapted virus strains in mammalian cells to be very potent against DENV 1-3. When tested in a mouse model, it has a modest effect (1 log reduction) on DENV1 viremia. In another completely different system, where HMAb 1C19 is added to DENV from patient sera and then tested for its inhibitory activity to infect mosquitoes [37], this showed HMAb 1C19 is not neutralizing. These observations suggest that HMAb 1C19 is not a good therapeutic agent, consistent with our findings. However, the results from this study have important implications for the design of therapeutic monoclonal antibodies for prevention or treatment of DENV infection.”

Reviewer #3: (No Response)

**Part III – Minor Issues: Editorial and Data Presentation Modifications**
Please use this section for editorial suggestions as well as relatively minor modifications of existing data that would enhance clarity.

Reviewer #1:  The concluding two paragraphs of the discussion really emphasize the rationale of this work and the potential therapeutic benefits. I would like to see that brought up in the introduction. Maybe some mention of dengue treatments and why antibody treatment is worth pursuing.

We have added the following into the first paragraph of introduction:

“Dengue virus (DENV), a flavivirus, infects about 390 million people each year [1]. Although there is a licensed vaccine available in some countries, its efficacy is low [2, 3], and there are no licensed antiviral drugs licensed to treat DENV infection [2-6]. DENV is spread through the bite of an infected *Aedes* mosquito [7]. Circulating DENV strains can be grouped into four serotypes (DENV1 to 4) [8, 9]. Disease associated with DENV infection ranges in severity from asymptomatic, to mild febrile illness, to life-threatening severe dengue characterized by hemorrhagic fever or shock. The difficulty in making a good vaccine against dengue is in large part due to the possibility of “antibody-dependent enhancement” (ADE) [10]. Although infection with one serotype elicits lifelong protection against that particular serotype, in a second infection with another serotype, the antibodies already present inside the patient may not be able to neutralize the second serotype leading to enhancement of disease severity. Therefore, a safe and effective vaccine will have to incorporate viruses from all serotypes, and the host recipient must elicit equally high protective immunity against DENV1-4. Testing with each serotype individually can elicit protective immune responses in the host, but when the four serotypes are combined together, the protective immune response in the recipient may not be equal. Also, after vaccination, the natural gradual decrease in antibody levels with time may also potentially lead to ADE. Therefore, development of an effective dengue vaccine has been extremely challenging. Furthermore, our previous results [11, 12] revealed that dengue virus can change its shape (smooth to bumpy surface or clubshape morphology) to escape from the immune system, and spontaneously undergo mutations that influence such shape changes, hence further complicating development of vaccines that may be prone to emergence of resistance. Therefore, it is important to investigate other possible therapies including antibody therapeutics. Here we study the effect of an antibody against different DENV morphologies.”

Reviewer #2: (No Response)

Reviewer #3: Figure 1:The authors should include scale bars in all cryoEM micrographs.

Scale bars have been included in all cryoEM micrographs on Figure 1.

Table 1 may also include the Kd value of 1C19 to E proteins to show its correlation with the IC50 of each strain.

Kd values of 1C19 to E proteins have been added into Table 1.

PLOS authors have the option to publish the peer review history of their article ([what does this mean?](https://journals.plos.org/plospathogens/s/editorial-and-peer-review-process#loc-peer-review-history)). If published, this will include your full peer review and any attached files.


If you choose “no”, your identity will remain anonymous but your review may still be made public.


**Do you want your identity to be public for this peer review?** For information about this choice, including consent withdrawal, please see our [Privacy Policy](https://www.plos.org/privacy-policy).

Reviewer #1: No

Reviewer #2: No

Reviewer #3: No

Figure Files:

While revising your submission, please upload your figure files to the Preflight Analysis and Conversion Engine (PACE) digital diagnostic tool, [https://pacev2.apexcovantage.com](https://pacev2.apexcovantage.com/). PACE helps ensure that figures meet PLOS requirements. To use PACE, you must first register as a user. Then, login and navigate to the UPLOAD tab, where you will find detailed instructions on how to use the tool. If you encounter any issues or have any questions when using PACE, please email us at [figures@plos.org](mailto:figures@plos.org).

Data Requirements:

Please note that, as a condition of publication, PLOS' data policy requires that you make available all data used to draw the conclusions outlined in your manuscript. Data must be deposited in an appropriate repository, included within the body of the manuscript, or uploaded as supporting information. This includes all numerical values that were used to generate graphs, histograms etc.. For an example see here: <http://www.plosbiology.org/article/info%3Adoi%2F10.1371%2Fjournal.pbio.1001908#s5>.

Reproducibility:

To enhance the reproducibility of your results, PLOS recommends that you deposit laboratory protocols in protocols.io, where a protocol can be assigned its own identifier (DOI) such that it can be cited independently in the future. For instructions see [http://journals.plos.org/plospathogens/s/submission-guidelines#loc-materials-and-methods](http://journals.plos.org/plospathogens/s/submission-guidelines)
